# Examining the Impact of Pro-Environmental Factors on Sustainable Consumption Behavior and Pollution Control

**DOI:** 10.3390/bs13020163

**Published:** 2023-02-14

**Authors:** Sania Khan, George Thomas

**Affiliations:** 1 Department of Human Resource Management, College of Business Administration, Prince Sattam Bin Abdulaziz University, Alkharj 11942, Saudi Arabia; 2 Department of Marketing, Prince Sultan University, Riyadh 11586, Saudi Arabia

**Keywords:** pro-environmental factors, pollution control, sustainable gasoline consumption, Saudi Arabian residents

## Abstract

Saudi Arabia is one of the most oil-rich countries in the world, and oil production is the country’s primary source of income. The aspects of greenhouse gas emissions and the harm they cause to the environment and residents have been overlooked because of the continuous emphasis on economic growth and a high reliance on oil resources. Consequently, environmental issues have become challenging for residents and questionable for industries. Despite various environmental awareness and pollution control studies around the world, Saudi Arabia’s pollution rate appears to be increasing. This study attempted to understand the impact of pro-environmental factors on pollution control and sustainable gasoline consumption in order to fill a research gap in the literature. Environmental awareness, self-efficacy and self-identity, ecological attitude, contextual factors, and social norms were considered as factors to investigate local residents’ sustainable consumption and pollution control behaviors. Primary data were collected from 416 local residents and analyzed using multiple regression. The results demonstrate the positive significant impact of pro-environmental variables on sustainable consumption and efforts toward pollution control. This study further presents practical implications for the Saudi government and environmentalists.

## 1. Introduction

The introduction of technology has greatly advanced the exploration of oil wells and their production. Johnston et al. [1] stated there will be a partial or full adverse effect on the overall environment. Though the positive sides of oil production include an increase in the national income and infrastructure, the negative side of its production is the pollution of the air, soil, and water. Therefore, the positive aspects are constantly focused on in oil production, ignoring the environmental issues. Many studies have reported the release of greenhouse gases (GHGs), with CO2 as the major polluting component, and subsequent health issues for humans [1,2,3,4]. However, Saudi Arabia lacks such studies. Saudi Arabia has been considered to be the country with the highest oil production in the world since the 1930s [5]. When oil reserves were discovered in Dammam, a city in the Eastern Province of Saudi Arabia, they sped up the city’s transformation from a tiny fishing community to a significant seaport. As a result, it serves as a hub for Saudi Arabia’s natural gas and petroleum industries. The city has an approximately 800 km^2^ area, with 1.279 million inhabitants as of 2021 and a per capita GDP of USD 23,352 in 2020 [6]. Middle Eastern countries produced only 11 million barrels per day during the 1980s and 15 million barrels in the 1990s, and they further increased their production capacity to 18 million barrels per day in the twenty-first century, with Saudi Arabia as a major contributor among them. With the realization of the increase in huge carbon emissions, Saudi Arabia executed green initiatives by establishing renewable sources for generating renewable and nuclear energy of up to 50 gigawatts (GW). Additionally, the latest price reforms on oil have enabled a reduction in oil consumption and pollution control [7]. Mazzetto et al. [8] demonstrated sustainable interventions toward improving Saudi tourism and how it will contribute to attaining the 2030 vision. Gonand et al. [9] assumed that the increase in local oil prices would improve the environment and reduce local oil consumption by promoting the local welfare of society and reinforcing oil exports. The strategic aim of the Saudi government toward the 2030 vision is to maintain low water, soil, air, and sound pollution [10]. The eco-friendly tree-planting program of Saudi Crown Prince Mohammed Bin Salman, post-disaster sustainable development, and sustainable building design are some of the environmentally friendly services that will help cities look attractive with an improved quality of living while consequently reducing the carbon footprint and global warming effect [11,12,13].

Since 2009, the consumption of oil resources has been increasing, and in 2018, consumption (in barrels per day) was the lowest. Due to extreme climatic conditions, the temperature reaches 50 degrees Celsius during summer and falls below 0 degrees Celsius in some regions during winter. So, to meet cooling requirements through air conditioners, most of the consumption of crude oil is predominantly used for the production of electricity, and the next largest consumer is the road transportation sector [14]. As a result, power consumption has increased tenfold during the last few decades, but the demand for gasoline in road transportation rose from 25 million barrels to 204 million barrels in 2015, which is an average of 6 percent per annum [15]. The Central Department of Statistics and Information (CDSI) expected population growth of roughly 30 million residents, which accounted for a 2.6% increase in 2014, and this may further increase the demand for gasoline required for residents to commute within the country. So, there will be potential growth in gasoline products with the increase in the population and, consequently, an intensification of carbon emissions. Alkhathlan et al. [15] examined the proportional link between carbon emissions and oil consumption by the transportation sector in Saudi Arabia and found that there is a notable increase in carbon emissions with an increase in the local population’s income. The most visited ten countries, namely, Turkey, China, the United States, Italy, France, Spain, Mexico, Thailand, the United Kingdom, and Germany, are prone to poorer environmental quality because of increased tourism activities [16].

In a similar vein, Almulhim et al. [17] made an effort to comprehend Saudi citizens’ environmental knowledge and attitudes toward the circular economy. According to the report, rather than being consciously conservative, the majority of Saudi Arabian citizens’ buying decisions are influenced by economic factors. The authors emphasized the vital importance of raising consumer awareness and giving them adequate information on trustworthy, environmentally friendly products. Hence, it is assumed that the awareness of environmental issues and consumption patterns is below the expected level among the Saudi population. Based on these assumptions, there is an immense need to fill this research gap to create a positive attitude toward environmental protection and sustainable consumption, and it is also essential to conduct more scientific studies in the Saudi Arabian context on the awareness of climate change and to understand its adverse effects on the ecosystem and sustainability. The research question lies in determining whether pro-environmental elements have an impact on local inhabitants’ sustainable consumption patterns, which in turn have an impact on pollution control. Therefore, the goal of this research project was to comprehend how Saudi citizens’ pro-environmental behaviors affect sustainable consumption, which in turn has an impact on pollution reduction with reference to the gasoline goods they use on a daily basis. Therefore, the purpose of this study was to bridge the knowledge gaps concerning Saudi Arabian residents’ pro-environmental behaviors, sustainable consumption, and pollution control. Consequently, the study’s key goals are (1) to comprehend how sustainable consumption is influenced by pro-environmental conduct and (2) to comprehend the connection between pollution prevention and sustainable consumption.

This study makes numerous significant contributions. Firstly, it expands on the body of evidence about Saudi citizens’ pro-environmental attitudes and efforts to reduce pollution in the nation. Second, the study results might help future researchers who look into other environmental subjects, such as how people utilize energy, air conditioners, and other equipment that runs on oil. Thirdly, it makes recommendations to the government for the formulation of oil consumption policies or the adoption of the appropriate green initiatives to transform the nation in accordance with Saudi Arabia’s 2030 vision. This article offers some inferences on the benefits of strengthening national public transportation, encouraging social life through green initiatives, and raising the standard of living in public areas, which can all contribute to improving the health of communities.

## 2. Literature Review

Saudi Arabia’s economic growth steadily increased after identifying oil sources and beginning oil production. Though the extant literature explains the adverse impact of oil production and consumption patterns on ecological development, there is a significant amount of ignorance among consumers about pro-environmental behavior and adherence to sustainable consumption patterns. Therefore, the literature is presented in two folds. Firstly, it provides the crude oil consumption patterns and gasoline demand and pricing policies in the last decades. Secondly, it presents the theoretical aspects of pro-environmental behavior that drive consumers toward sustainable consumption and pollution control.

### 2.1. Crude Oil Consumption Pattern in Saudi Arabia

Among the countries in the world, Saudi Arabia is one of the geographical locations that face extremely hot climatic conditions. Especially during summer, there is a high demand for electricity consumption among industries and residents due to the increase in the use of air-conditioning systems. According to the Joint Organizations’ Data Initiative (JODI), the nation burned a huge amount of crude oil, 0.9 million barrels per day, during the month of July. It was recorded as the highest consumption of crude oil since August 2010. In comparison, the consumption of crude oil from 2009 to 2013 for power generation was 0.7 million barrels per day. During the same period, two other Middle Eastern countries, Kuwait and Iraq, ranked first and second in crude oil consumption, with approximately 0.08 million barrels per day. While most countries depend on consuming natural gas or coal for electricity production, the nation suffers from the non-availability of local coal production. On the other hand, it lacks such natural gas production operations due to the large amount of sulfur content in natural gas and the low price of domestic natural gas. Hence, there were no further lucrative foreign direct investments (FDIs) to be found in natural gas ventures [18]. During the same decade, in 2012, the total electricity consumption doubled from that in 2000 to roughly 232 billion kWh. However, this did not pause the nation’s economic growth. Rather, it boosted it to 4.7% in 2014 from 3.8% in 2013. The Central Department of Statistics and Information (CDSI) predicted the growth of the population at roughly 30 million inhabitants, accounting for a 2.6% increase in 2014, which may still increase the electricity demand [18]. The sustainability initiative taken by the Saudi government focused on diversifying the sources of power generation and adopted more energy-efficient methods to increase the generation capacity from 58 gigawatts (GW) to 120 GW by 2032 using renewable energy sources such as solar and nuclear power. This approach reduces dependence on crude oil, except for the production of more diesel [19].

**Figure 1:**
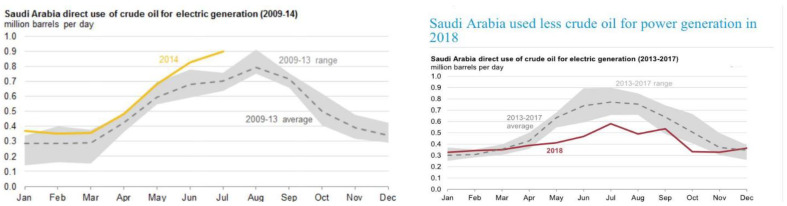
Crude oil consumption from 2009 to 2018. Source: U.S. Energy Information Administration.

**Figure 2:**
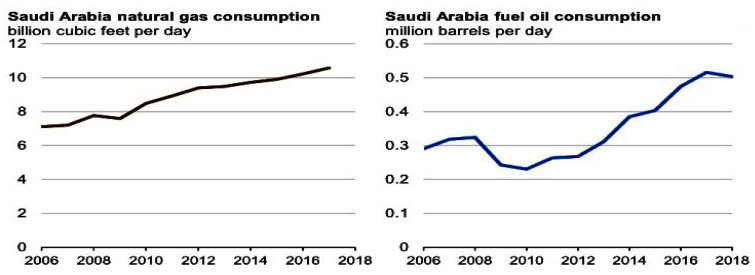
Comparative consumption of oil and natural gases. Source: U.S. Energy Information Administration.

Despite the objective of reducing crude oil consumption, the highest record of burning crude oil for power generation was again seen in 2015, with 0.9 million barrels per day. However, as committed, Saudi Arabia achieved a controlled consumption of less crude oil in 2018 for electricity generation at an average of 0.4 million barrels per day, which was in fact much less than in 2009. This was because of the parallel use of fuel oil and natural gases. The comparative usage of crude oil for power generation from 2009 to 2014 and 2013 to 2018 is illustrated in Figure 1. Natural gases are produced in association with the wells of crude oil production, though these wells are not connected with regular oil production. Hence, the consumption of natural gases also apparently increased [20]. Figure 2 shows the comparative consumption of both oil and natural gases.

### 2.2. Gasoline Demand and Pricing Policy

During the last few decades, the consumption of gasoline increased by 6 percent per annum, and the consumption per capita is derived from the ratio of total gasoline consumption by the population in the country.

Gasoline consumption per capita = Gasoline consumption/Population in KSA.

From 2010 to 2018, consumption increased from 2.44 L per capita to 3.07 L per capita. Below, Table 1 illustrates more details on the indicators during the respective years. In 2015, the Saudi government increased the price of water, fuel, and electricity for domestic consumption, and the price of gasoline increased from SAR 0.45 to SAR 0.60 for 91 octane grade and from SAR 0.75 to SAR 0.90 for 95 octane grade [21]. However, the price has changed multiple times during the course of time, and the current rates in the year 2021 for 91 and 95 octane grades are SAR 2.18 and SAR 2.33, respectively. Due to the increase in prices, the estimation of demand and supply curves demonstrated that the improvement in social welfare would be approximately SAR 1 to 2 billion per annum in 2010, which amounts to 0.1 percent of GDP in 2015. There are no exact studies that measured the carbon emissions in relation to the amount of gasoline consumption. The study conducted by Alkhathlan et al. [15] attempted to evaluate the impact of total oil consumption and transportation on Saudi Arabia’s environment. However, the estimation of carbon emissions from the underlying energy demand trend (UEDT) and the transportation sector did not consider the gasoline demand, nor price and income variations.

### 2.3. Antecedents of Pro-Environmental Consumer Behavior

Following the late 1960s’ ecological activism, environmental concerns began to play an increasing role in anti-sprawl critiques. According to this school of thought, moderate settlement led to substantial energy consumption, particularly through automobile access. This, in turn, tends to result in resource depletion and increased pollution. The reduced settlement also resulted in the loss of grassland, more health issues among citizens, and ozone layer depletion. Attempts to promote environmentally friendly consumption are frequently grouped under the umbrella term “sustainability”. Evidently, energy consumption in Arab countries is significantly greater than in other countries. Many ecologists believe that this is a universal human challenge that can only be overcome through strict mitigation measures and metropolis redesign to be more energy-efficient. The huge usage of personal vehicles is the most acrimonious area involving both gasoline consumption and the emission of carbon [22,23,24].

Various influencing factors have been identified in research studies on understanding consumer behavior and sustainable consumption. The majority of these studies found that pro-environmental factors influenced consumers’ attitudes toward sustainable consumption and pollution control. Some of these pro-environmental behavioral factors were also presented in the theory of planned behavior (TPB). For example, social norms were defined as consumer expectations from a group of individuals about a specific behavior and as a foundation for how people choose to adapt their behaviors and attitudes in relation to the social context. The personal norm is the moral commitment of the consumer to behave ecologically in a given situation. Similarly, consumer attitudes toward the environment were discussed in multiple research studies [25,26,27,28].

#### 2.3.1. Environmental Awareness

As stated by Roy [29], environmental consciousness and awareness are related to cognitive factors, which comprise knowledge, memory, thinking, and the mitigation of problems. Consumer responsibility for universal ecological conditions and the desire to improve them are also associated with this factor. The greater their awareness, the more they are engaged in sustainable consumption [30,31,32,33]. Kollmuss et al. [34] illustrated the difference between ecological knowledge about facts and their actions. Information about facts is related to environmental problems and their causes and consequences, and information about actions is associated with environmentally friendly consumption. This statement was also statistically validated by Tanner et al. [35]. Based on this concept, the following is hypothesized.

**Hypothesis** **1** **(H1):** *Consumer environmental awareness has a significant positive influence on sustainable consumption.*

#### 2.3.2. Self-Efficacy and Self-Identity

The construct of self-efficacy is the ability of consumers to engage in a prospective situation, get motivated, and rectify their actions while facing challenges. Such individuals feel their contribution may help solve global problems [36]. Janmaimool et al. [25] also asserted that self-efficacy encourages pro-environmental engagement and sustainable consumption. On the other hand, self-identity is characterized by what individuals believe about themselves in the context of being environmentally conscious. Such consumers were found to behave sustainably and tend to use sustainable transportation [37]. It is evident that pro-environmental self-identity has a significant positive effect on sustainable consumption. Therefore, the following is hypothesized.

**Hypothesis** **2** **(H2):** *Consumer pro-environmental self-efficacy and self-identity have a significant positive influence on sustainable consumption.*

#### 2.3.3. Ecological Attitude

This behavioral factor is associated with consumers who are positive and concerned about environmental protection in all aspects of their lives, as well as have optimistic and pessimistic opinions and responses to individuals, objects, and circumstances [38]. Various other key components, such as environmental perception, personal norms, values, and connection to place, are included in this factor [25,39]. As stated by Schwartz et al. [28], there are two types of consumer values: self-transcendence and self-enhancement. Self-transcendence consumers are those who are strongly inspired by qualities such as social responsibility, open-mindedness, advisableness, and truth; self-enhancement consumers are those who are concerned about society and devote less time to personal needs. Personal norms, as a sub-factor of ecological attitude, influence consumers’ pro-environmental behaviors and promote environmentally friendly consumption. Previous studies [40,41,42] demonstrated a positive relationship between personal norms and pro-environmental behavior during sustainable consumption. Connection to place is correlated with better emotional reactions, and such consumers are more inclined to protect the environment around them and promote ecological consumption [43]. Hence, the following is hypothesized.

**Hypothesis** **3** **(H3):** *Consumers’ ecological attitude has a significant positive influence on sustainable consumption.*

#### 2.3.4. Contextual Factors

These are the external factors that influence consumers’ sustainable consumption. Such conditions exist during the purchase, use, and disposal of goods and services [44]. In this context, Wang et al. [45] investigated consumers’ decisions to use public transportation and wastewater treatment; Maki et al. [46] examined energy conservation behavior and stated that the prices of various commodities and services are associated with positive and negative thoughts concerning sustainable consumption. Therefore, consumers adjust their behavior according to the situation. Therefore, the following is hypothesized.

**Hypothesis** **4** **(H4):** *Contextual factors have no significant influence on sustainable consumption.*

#### 2.3.5. Social Norms

These norms include the common beliefs, basic rules, and perspectives of society, as well as the acceptance of a particular behavior by an individual and the public in general [47]. When consumers are new and have little experience with certain goods and services, this factor has a strong influence. Such behavior is commonly seen among family members and relatives. So, the following is hypothesized.

**Hypothesis** **5** **(H5):** *Social norms have a significant positive influence on sustainable consumption.*

### 2.4. Sustainable Consumption and Pollution Control

Sustainable consumption refers to people committing to using minimum products, services, and natural resources in order to live a quality lifestyle while also protecting the environment by reducing toxic substances, carbon emissions, and other pollutants. It also emphasizes meeting current needs while also sustaining future needs. As reported by OECD studies, there is a significant difference in sustainable consumption patterns by age. Young people realize their consumption patterns have an adverse impact on society and the environment. They are found to be more ecologically conscious in reducing pollution and contributing to the development of human health [48]. According to a study conducted by Parry et al. [49], including the gasoline consumption pattern of Saudi Arabia, the price inflation in gasoline minimizes the gasoline consumption of passengers and considers the overall travel distance traveled by them. This approach also assists in decreasing hazardous gas emissions and air pollution and reduces road accidents and congestion. Based on this, the following is hypothesized.

**Hypothesis** **6** **(H6):** *Sustainable consumption has a significant positive influence on sustainable consumption.*

## 3. Research Methodology

**Figure 3:**
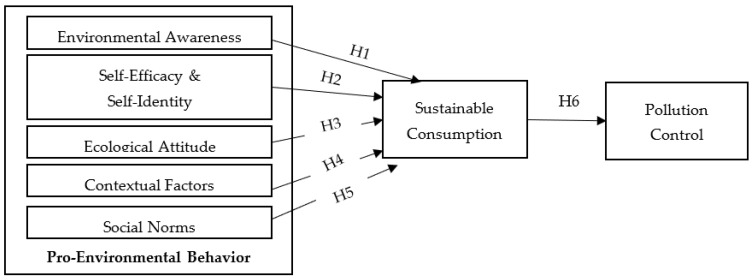
Conceptual framework of pro-environmental behavior, sustainable consumption, and pollution.

Based on the formulated research hypotheses, the research framework of the study was conceptualized as shown in Figure 3.

This study employed a self-administered questionnaire with closed-ended questions. For the constructed items, questions were developed on a 5-point Likert scale for the study, with 5 as strongly agree, 4 as agree, 3 as neutral, 2 as disagree, and 1 as strongly disagree. A simple random sampling method has been used to distribute the questionnaires online by email and sharing the link to a Google Form among male and female residents of Saudi Arabia within the age group of 20 to 50 years who are employed and salaried in a public or private organization and commute using their own automobile. The simple random sampling method gives all individuals in the population an equal chance of being selected for the sample. According to Meyer [50], a higher level of education stimulates individuals to care about social welfare and act in an environmentally responsible way. So, the respondents’ level of education is one of the important demographic criteria for interpreting the research findings for a variety of reasons. First and foremost, respondents will be able to comprehend the proper definition of pro-environmental variables, and this understanding will tremendously help in converting their thoughts and expressions into opinions while filling in the survey questionnaire. Second, information was gathered at random from Saudi workers at various occupational levels. Therefore, a bachelor’s degree or higher was the minimum educational level considered in this study for the data collection. The questionnaire yielded a total of 416 valid responses, which were analyzed further in SPSS using multiple regression.

## 4. Data Analysis and Results

This study used multiple regression analysis to explore the underlying dimensions of the pro-environmental, sustainable consumption, and pollution control constructs and further tested the formulated hypotheses. Below, Table 2 demonstrates the means, factor loadings, and reliability conditions of the collected data. To determine the underlying dimensions, the exploratory factor analysis (EFA) method with principal component analysis (PCA) with varimax rotation was used and revealed the acceptability of all factor loadings, which are above 0.6. The factor loadings of EA are from 0.790 to 0.981 with an alpha value of 0.806; those of SEI are from 0.765 to 0.874 with an alpha value of 0.841; those of EAT are from 0.802 to 0.900 with an alpha value of 0.748; those of CF are from 0.769 to 0.938 with an alpha value of 0.782; those of SN are 0.850 to 0.900 with an alpha value of 0.841; those of SC are 0.771 to 0.882 with an alpha value of 0.943; and those of PC are 0.974 to 0.980 with an alpha value of 0.0.854. Additionally, the internal consistency (Cronbach alpha) values are all in a tolerable range above 0.7 and verify the reliability condition [51]. The factorability conditions were also confirmed by a Kaiser–Meyer–Olkin value of 0.832 and a significant Bartlett’s test of sphericity (*p* < 0.001).

The results of multiple regression are shown in Table 3, Table 4, Table 5 and Table 6. The summary of the regression analysis for the sustainable consumption of gasoline automobiles is shown in Table 3. According to the regression model, the R-square is 0.985, indicating that 98.5 percent of the factor variance contributes to sustainable consumption.

Table 4 presents the regression model coefficients, which show that the independent variables EA, SEI, EAT, CF, and SN are significant and explain Saudi residents’ sustainable consumption. H1, H2, H3, H4, and H5 are supported based on these findings. It is also clear that self-efficacy and self-identity have the least influence on sustainable consumption, while ecological attitudes have the most influence. Hence, it is widely assumed that residents possess a good attitude toward the environment. However, it is observed that the recent increase in gasoline prices has pushed residents toward more environmentally friendly consumption.

The summary of the regression analysis for pollution control is shown in Table 5. According to the regression model, the R-square is 0.888, indicating that 88.8 percent of the factor variance contributes to pollution control. Table 6 represents the regression model coefficients of PC and demonstrates that the independent variable SC has a significant influence on PCs and explains Saudi residents’ sustainable consumption, leading to pollution control. Hence, H6 is supported.

## 5. Discussion

In order to investigate the underlying and influential factors of Saudi Arabian consumers’ sustainable consumption, the current study sought to examine the relationship between Saudi Arabian consumers’ pro-environmental behaviors, sustainable consumption, and pollution control. All of the survey participants are educated, currently residing in Saudi Arabia, conscious of sustainable consumerism, and aware of the damaging effects of pollution on the environment. The study’s findings demonstrate that pro-environmental consciousness is the cornerstone of sustainable consumerism. It is challenging to adopt sustainable consumption if the person lacks pro-environmental consciousness. Environmental awareness, self-efficacy and self-identity, ecological attitude, contextual factors, and social norms are among the five pro-environmental characteristics that all significantly influence sustainable consumer behavior. These findings are in line with those of [52,53,54]. The majority of participants expressed worry about pollution control and agreed that it was each person’s responsibility to reduce excessive gas consumption. The outcomes additionally showed how sustainable consumption has a beneficial effect on pollution reduction. Even though gasoline is relatively inexpensive in Saudi Arabia, respondents are aware of the harm that excessive usage does to the environment. As a result, they favor purchasing eco-friendly cars and, where possible, using public transportation. However, examining pro-environmental behaviors and attitudes alone cannot be used to gauge how much gasoline use among consumers shifts to a more sustainable level. It is more crucial to concentrate on a variety of other aspects of sincere intentions that motivate sustainable or ecological actions, such as the extent of this shift in consumer-used car models and types, the yearly average distance driven by standard-sized cars, the switch from older to newer environmentally friendly models, and the implications of switching between different car models. The survey confirmed that the ongoing rise in gas prices will undoubtedly have a favorable effect on Saudi citizens’ consumption patterns and even raise awareness among those who do not currently act sustainably. According to the report, the overall findings of the study are clearly supported by the Saudi Arabian government’s current green efforts, which it is swiftly putting into action in line with its 2030 goal [10].

## 6. Conclusions

The purpose of this study was to assess the pro-environmental behaviors of Saudi residents in terms of their sustainable consumption of gasoline through the use of automobiles and its effect on pollution control. This study demonstrated a positive influence of pro-environmental behavioral factors on sustainable consumption and a further significant impact on pollution control. However, the measures of the constructs revealed that the residents’ actions are not as strongly reflective of their ecological knowledge. Most respondents agreed that they travel by car when necessary rather than taking public transportation because public transportation, such as metro trains and buses, is frequently not connected to their destination. In addition, they feel more at ease and save money when they travel in their own vehicles. They are more influenced by environmental attitudes when purchasing new automobiles. They may also change their minds based on other factors, namely, cost, comfort, necessity, and location. Though the relationship between the independent variables and the dependent variables is significant, it is assumed that such behavior has recently been observed following the increase in gasoline prices. Such fluctuating gasoline consumption patterns and reduction in pollution were also commonly noted by past researchers, both within Saudi Arabia and in other countries [18,55].

However, the extent to which consumers’ gasoline consumption shifts to a more sustainable level cannot be determined solely by examining pro-environmental behaviors and attitudes. It is more important to focus on many other aspects of genuine intentions that drive sustainable or ecological actions, such as the degree of the shift in the models and types of cars used by consumers, the annual average distance traveled by standard-sized cars, the transition from old models to new eco-friendly automobiles, and the implications of shifting between various automobile models. Furthermore, the study made no attempt to understand whether the reduction in pollution and improvement in fuel consumption were the result of a reduction in fuel consumption or a decrease in the annual average travel distance. Additionally, the study made no specific attempt to investigate other modes of gasoline consumption, such as the consumption of diesel by daily logistics for commercial purposes, households, etc. Furthermore, the results are based on the respondents’ ecological knowledge and attitudes toward sustainable consumption. The real intentions leading to their actual actions were not investigated. So, additional studies with a larger sample size are required to confirm the findings of this study, along with other means of gasoline consumption patterns.

As a result, before expecting the sustainable consumption of gasoline or efforts to reduce pollution control, the government must provide adequate public transportation at an affordable cost to citizens and residents. Furthermore, the government can use a few tools, such as ecological awareness campaigns, subsidies and taxes, environmental standards, and stringent regulations to use light gasoline-powered vehicles, to encourage residents to consume gasoline in a more sustainable manner and to reduce pollution.

## Figures and Tables

**Table 1:** Per capita consumption of gasoline, 2010–2018 (Source: General Authority for Statistics).

Years	Indicator		
	Gasoline Consumption	Population in Saudi Arabia	Consumption of Gasoline per Capita
	Unit		
	Thousand Tons	Number	Liter per Capita
2010	18,114	27,410,510	**2.44**
2011	19,172	28,173,195	**2.52**
2012	20,783	28,896,842	**2.65**
2013	21,702	29,613,068	**2.71**
2014	22,659	30,339,797	**2.76**
2015	24,355	31,062,072	**2.9**
2016	29,032	31,787,580	**3.37**
2017	29,694	32,612,846	**3.37**
2018	27,765	33,413,660	**3.07**

**Table 2:** Means, factor loadings, and reliability values of pro-environmental behavior toward automobiles’ sustainable gasoline consumption and pollution control (Source: Author).

	Dimensions and Items	Mean	Factor Loading	Cronbach Alpha Value
Environmental Awareness (EA)		3.59		0.806
	I am aware that my use of automobiles will result in carbon footprints and environmental pollution (EA1).		0.875	
	I believe it is ones’ responsibility to put down excessive consumption of gasoline for controlling pollution (EA2).		0.981	
	I recall my ecological knowledge while driving and strictly adhere to sustainable gasoline consumption (EA3).		0.802	
	My environmental actions are reflective of my ecological knowledge (EA4).		0.790	
Self-efficacy and Self-identity (SEI)		2.79		0.841
	I engage a lot of time thinking about how much damage overconsumption causes to the environment that we live in (SEI1).		0.874	
	I travel only when necessary and forego my pleasures to contribute to a better environment (SEI2).		0.831	
	I believe I am consuming fewer gasoline products and promote sustainable transportation (SEI3).		0.870	
	During my holidays, I stay at home and give up the pleasure of visiting places to practice sustainable consumption (SEI4).		0.765	
Ecological Attitude (EAT)		2.81		0.748
	I care to buy environmentally friendly automobiles (EAT1).		0.900	
	I am a socially responsible individual who is eager to educate others about the environmental impact of gasoline products (EAT2).		0.886	
	A pollution-free atmosphere motivates me to sustain the future environment (EAT3)		0.802	
	Since I care about the environment, I take a shared car to work (EAT4).		0.877	
Contextual Factors (CF)		3.14		0.782
	I am willing to pay a high price for environmentally friendly automobiles (CF1).		0.771	
	I am willing to use public transportation because I want to live a low-carbon lifestyle (CF2).		0.769	
	I believe that the recent constant increase in gasoline prices will have a positive impact on Saudi residents’ sustainable consumption (CF3).		0.938	
	Travelling by own car is much cheaper and more comfortable for me than taking public transportation (CF4).		0.880	
Social Norms (SN)		2.83		0.841
	I have recommended my family, friend, and colleagues at work not to buy some automobiles which emit high carbon pollutants (SN1).		0.860	
	When I buy a new car, I ask my friends for advice on the mileage (SN2).		0.900	
	There is frequent public transportation in Saudi Arabia, such as metro trains and national buses that connect to various locations (SN3).		0.850	
Sustainable Consumption (SC)		3.27		0.943
	I choose automobiles that are non-polluting and friendly to the environment SC1).		0.882	
	I understand the true meaning of sustainability and practice environmentally friendly usage of gasoline vehicles (SC2).		0.771	
	I am pleased when my own gasoline consumption choices contribute to pollution control (SC3).		0.880	
Pollution Control (PC)		2.75		0.854
	I believe in practicing sustainable consumption, and the same can contribute to pollution control (PC1)		0.974	
	Even in the future, with the proportionate rise in the size of Saudi residents, the overall pollution from gasoline can be controlled through sustainable consumption (PC2).		0.980	

**Table 3:** Model summary of SC (Source: Author).

Model	R	R^2^	Adjusted-R^2^	F	Sig
**1**	0.985 ^a^	0.970	0.969	1053.508	0.000

R^2^ = coefficient of regression; R = regression value; F = degree of freedom; sig = significance. ^a^. Predictors: (Constant), EA, SEI, EAT, CFI, SN.

**Table 4:** Coefficients—model summary of SC (dependent variable: SC) (Source: Author).

Model	Unstandardized Coefficients		Standardized Coefficients	t-Value	Sig.
	β	Std. Error	Beta		
**(Constant)**	9.501	1.985		3.851	0.005
**EA**	0.795	0.179	0.261	2.574	0.035
**SEI**	0.204	0.049	0.254	4.152	0.000
**EAT**	0.617	0.135	0.697	4.566	0.000
**CF**	0.319	0.057	0.377	5.561	0.000
**SN**	0.341	0.113	0.436	3.007	0.003

β = beta; t = *t*-test value; sig = significance.

**Table 5:** Model summary of PC (Source: Author).

Model	R	R^2^	Adjusted-R^2^	F	Sig
**1**	0.888 ^a^	0.789	0.787	619.814	0.000

R^2^ = coefficient of regression; R = regression value; F = degree of freedom; sig = significance. ^a^. Predictors: (Constant), SC.

**Table 6:** Coefficients—model summary of PC (dependent variable: PC) (Source: Author).

Model	Unstandardized Coefficients		Standardized Coefficients		
	β	Std. Error	β	t	Sig.
**(Constant)**	0.651	0.086		7.593	0.000
**SC**	0.639	0.026	0.888	24.896	0.000

β = beta; t = *t*-test value; sig = significance.

## Data Availability

Not applicable.
